# Inhibition of store-operated channels by carboxyamidotriazole sensitizes ovarian carcinoma cells to anti-Bclx_L_ strategies through Mcl-1 down-regulation

**DOI:** 10.18632/oncotarget.26084

**Published:** 2018-09-21

**Authors:** Marie-Laure Bonnefond, Romane Florent, Sophie Lenoir, Bernard Lambert, Edwige Abeilard, Florence Giffard, Marie-Hélène Louis, Nicolas Elie, Mélanie Briand, Denis Vivien, Laurent Poulain, Pascal Gauduchon, Monique N'Diaye

**Affiliations:** ^1^ Normandie University, UNICAEN, INSERM U1086 ANTICIPE, Interdisciplinary Research Unit for Cancer Prevention and Treatment, BioTICLA Axis, Biology and Innovative Therapeutics for Ovarian Cancers, Caen, France; ^2^ UNICANCER, François Baclesse Cancer Center, BioTICLA Laboratory, Caen, France; ^3^ Normandie University, UNICAEN, INSERM UMR-S 1237, Physiopathologie et Imagerie des Troubles Neurologiques (PhIND), tPA and Neurovascular Disorders Team, Caen, France; ^4^ Délégation Régionale de Normandie, CNRS, Caen, France; ^5^ Normandie University, UNICAEN, Centre de Microscopie Appliqué à la Biologie, CMabio3, Structure Fédérative 4206 ICORE, Caen, France; ^6^ Centre de Ressources Biologiques, OvaRessources, François Baclesse Cancer Center, Caen, France

**Keywords:** ovarian cancer, Store-operated calcium channels, mTORC1, MCL-1, ABT-737

## Abstract

The anti-apoptotic proteins Bcl-x_L_ and Mcl-1 have been identified to play a pivotal role in apoptosis resistance in ovarian cancer and constitute key targets for innovative therapeutic strategies. Although BH3-mimetics (i.e. ABT-737) potently inhibit Bcl-x_L_ activity, targeting Mcl-1 remains a hurdle to the success of these strategies. Calcium signaling is profoundly remodeled during carcinogenesis and was reported to activate the signaling pathway controlling Mcl-1 expression. In this context, we investigated the effect of carboxyamidotriazole (CAI), a calcium channel inhibitor used in clinical trials, on Mcl-1 expression. CAI had an anti-proliferative effect on ovarian carcinoma cell lines and strongly down-regulated Mcl-1 expression. It inhibited store-operated calcium entry (SOCE) and Mcl-1 translation through mTORC1 deactivation. Moreover, it sensitized ovarian carcinoma cells to anti-Bcl-x_L_ strategies as their combination elicited massive apoptosis. Its effect on mTORC1 and Mcl-1 was mimicked by the potent SOCE inhibitor, YM58483, which also triggered apoptosis when combined with ABT-737. As a whole, this study suggests that CAI sensitizes to anti-Bcl-x_L_ strategies *via* its action on Mcl-1 translation and that modulation of SOCE could extend the therapeutic arsenal for treatment of ovarian carcinoma.

## INTRODUCTION

Despite its low incidence, epithelial ovarian carcinoma (EOC) is the fifth-leading cause of cancer-related deaths in women. It was estimated that in 2017, there would be more than 22240 new cases of ovarian cancers and 14080 deaths in the United States [1]. The standard treatment consists of wide-margin surgical resection and a platinum-based chemotherapy in combination with paclitaxel [2]. Despite a good response to first-line chemotherapy, 80% of patients relapse during the first 18 months due to chemoresistance. Escape from apoptosis is one of the major hallmarks of cancer cells and is frequently due to alteration of the ratio of [anti- versus pro-apoptotic] members of the Bcl-2 family, which leads to an increase in apoptotic threshold during oncogenic stress [3]. This altered ratio could be a therapeutic opportunity and it is widely accepted that targeting anti-apoptotic members is a relevant strategy to induce apoptosis in many types of cancer [4]. Our group previously showed that the anti-apoptotic proteins Mcl-1 and Bcl-x_L_ were able to cooperate to allow ovarian carcinoma cells to overcome apoptosis, as their concomitant inhibition was sufficient to induce cell death [5–7]. These proteins are now considered as relevant targets for the treatment of chemoresistant ovarian cancers, and the identification of adequate strategies for their efficient inhibition constitutes an exciting challenge.

The crucial role of Bcl-x_L_ in cancers has led to the development of many tools to counteract its anti-apoptotic effect such as BH3-mimetics. These molecules, which mimic the BH3 domain of pro-apoptotic proteins, can hinder the function of anti-apoptotic proteins by binding their hydrophobic groove. Among these molecules, ABT-263 (Navitoclax), the orally administrable derivative of ABT-737, is currently undergoing phase I and II clinical trials in different locations including ovarian cancer (NCT02591095). However, ABT-737 does not target Mcl-1 and overexpression of this protein constitutes a hurdle to ABT-737-induced apoptosis [6].

Several strategies have been tested to inhibit Mcl-1 expression or activity. Among these, the benefit of PI3K/Akt/mTOR inhibitors to control Mcl-1 expression was investigated. Targeting this survival pathway is a key objective for gynecological cancer therapy as it is overactivated in almost half of high-grade serous ovarian tumors. Moreover, mTORC1 is known to control Mcl-1 translation through 4E-BP1 phosphorylation [8–13]. In this context, the combination of PI3K/Akt/mTOR inhibitor with ABT-737 induced apoptosis in various cancer types including ovarian cancers *in vitro* and *in vivo* PDX models [14–17].

Among the different possibilities to impede PI3K/Akt/mTOR activation, the role of calcium has been under study for several years and is particularly attractive. Calcium is the most important second messenger in the cell and it regulates fundamental physiological events such as gene expression, survival and cell death. Its impact on cell fate depends on the fine regulation of the amplitude and/or frequency of its signal [18–21]. As cancer cells require intense metabolism for their growth and motility, carcinogenesis often occurs with the modulation of calcium homeostasis (via modulation of calcium channels and pumps) for supplying cancer cells and activating pro-survival pathways [21–23]. Several studies have shown that mTORC1 is a target for calcium [24–31]. Recently, we showed that calcium chelation by BAPTA-AM and calmodulin inhibition by W7 led to a decrease in Mcl-1 *via* down-regulation of the mTORC1/4E-BP1 pathway and sensitized ovarian cancer cells to anti-Bcl-x_L_ strategies [13].

Modulating calcium signaling is now viewed as an emerging anti-tumoral strategy but only a few calcium inhibitors have been included in clinical trials to date [20, 21]. One of them, carboxyamidotriazole (CAI), was shown to have anti-tumoral and anti-angiogenic properties *in vitro* and *in vivo* through its ability to inhibit calcium channels such as Store-Operated Calcium Channels (SOC) [32–40]. CAI and its pro-drug salt form (carboxyamidotriazole orotate - CTO) have reached several clinical trials in various solid cancers including ovarian carcinoma, cervical cancer, renal cell carcinoma, melanoma or glioblastoma [41–48]. Reported results showed that CAI used as a single agent or in combination with paclitaxel or temozolomide has a well-tolerated toxicity profile with low grade side-effects such as fatigue, nausea or reversible peripheral neuropathy. CAI exhibited mild anticancer properties in some clinical trials, however it was described to stabilize 31% of patients with relapsed ovarian cancer for more than 6 months and its combination with Temozolomide displayed effective antitumor activity in glioblastoma [45, 48]. As we previously showed that Mcl-1 is a target for calcium signaling, we investigated whether CAI could modulate the expression of Mcl-1, with a special attention to the molecular mechanism involved and whether it could sensitize platinum-refractory ovarian cancer cells to anti-Bcl-x_L_ strategies.

## RESULTS

### CAI inhibits Mcl-1 expression and has an anti-proliferative effect on ovarian carcinoma cells

**Figure 1:**
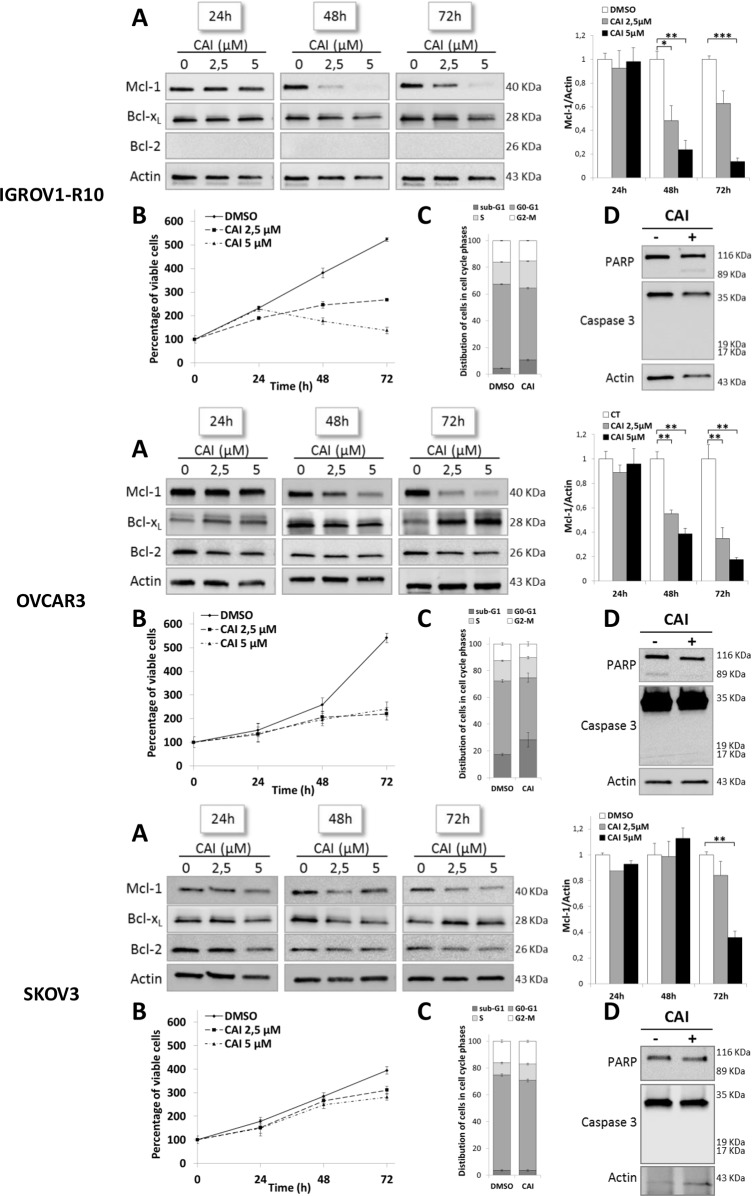
CAI inhibits Mcl-1 protein expression and has an anti-proliferative effect on three ovarian cell lines **(A)** Expressions of Mcl-1, Bcl-x_L_ and Bcl-2 were assessed by western blot in IGROV1-R10, OVCAR3 and SKOV3. Cells were treated by increasing concentrations of CAI for 24h, 48h and 72h. Mcl-1 protein expression upon CAI treatment in the three cell lines tested was quantified with Image J software. Data are expressed as mean ± SEM of three independent experiments. Statistical differences were analyzed with a Student t-test: ^*^p< 0.05, ^**^p< 0.01, ^***^p< 0.001 (n=3). **(B)** Number of viable cells was assessed by blue trypan exclusion. Curves show the percentage of viable cells normalized to the number of viable cells at the beginning of treatment (100%). Results are expressed as mean ± SEM of three independent experiments (n=3). **(C)** Histograms represent the distribution of cells in cell cycle phases (sub-G1, G0-G1, S and G2-M) induced by 5 μM CAI for 48h (IGROV1-R10 cells) and 72h (for OVCAR3 and SKOV3 cells) and studied by flow cytometry. Results are expressed as mean ± SEM of three independent experiments. **(D)** The effect of 5μM CAI on PARP and caspase 3 cleavages was assessed by western blot for 24h, 48h and 72h in IGROV1-R10, OVCAR3 and SKOV3 cell lines.

The expression of the Bcl-2 family anti-apoptotic members was analyzed in IGROV1-R10, OVCAR3 and SKOV3 cell lines treated with increasing concentrations of CAI from 24h to 72h. Whereas no variation in Mcl-1 expression was noticed in the three cell lines after 24h of treatment, a drastic decrease was observed from 48h of treatment in IGROV1-10 and from 72h of treatment in OVCAR3 and SKOV3 cells (Figure 1A). This decrease appeared from 2.5 μM of CAI and was accentuated for 5 μM. Regarding the other anti-apoptotic members, Bcl-x_L_ expression was not down-regulated by CAI and was instead slightly induced after 72h of treatment in OVCAR3 and SKOV3, but not IGROV1-R10 cells (Figure 1A). Bcl-2 was not expressed in IGROV1-R10 cells as previously described [13] and was not significantly modulated upon CAI treatment for OVCAR3 and SKOV3 (Figure 1A).

The study of the impact of CAI on these cell lines proliferation showed that from a concentration of 2.5 μM, CAI exerted an anti-proliferative effect which was observed after 48h in IGROV1-R10 cells and after 72h in OVCAR3 and SKOV3 cells (Figure 1B). Nevertheless, it was not accompanied by a significant blockade in cell cycle phases. In conditions leading to Mcl-1 inhibition (5μM / 48h for IGROV1-R10, 5μM / 72h for OVCAR3 and SKOV3), CAI did not induce apoptosis, a finding confirmed by the absence of sub-G1 peak and PARP and caspase 3 cleavages (Figure 1C and 1D). Therefore, CAI-induced Mcl-1 inhibition is not a consequence of cell death or cleavage by caspase 3 [49]. Moreover, the absence of cell death could be ascribed to Bcl-x_L_ maintenance in response to CAI.

### Store-mediated Ca^2+^ entry is inhibited in ovarian carcinoma cell lines by CAI treatment

As CAI is known to inhibit various calcium channels, we attempted to identify the nature of the channels it targets in ovarian carcinoma models [32–37]. For this purpose, store-operated calcium entry (SOCE) was evaluated by using “Ca^2+^ re-addition” methodology [50]. SOCE is an ubiquitous extracellular Ca^2+^ entry pathway. Upon calcium RE depletion by IP3 receptor (IP3R) opening, the Ca^2+^ sensor molecules STIM-1 (Stromal Interaction Molecule-1) aggregate on the RE surface and physically interact with ORAI 1 proteins (Calcium release-activated calcium channel protein 1) located on the plasma membrane. This leads to the formation of a store-operated channel (SOC) that allows calcium influx from the extracellular medium [51].

**Figure 2:**
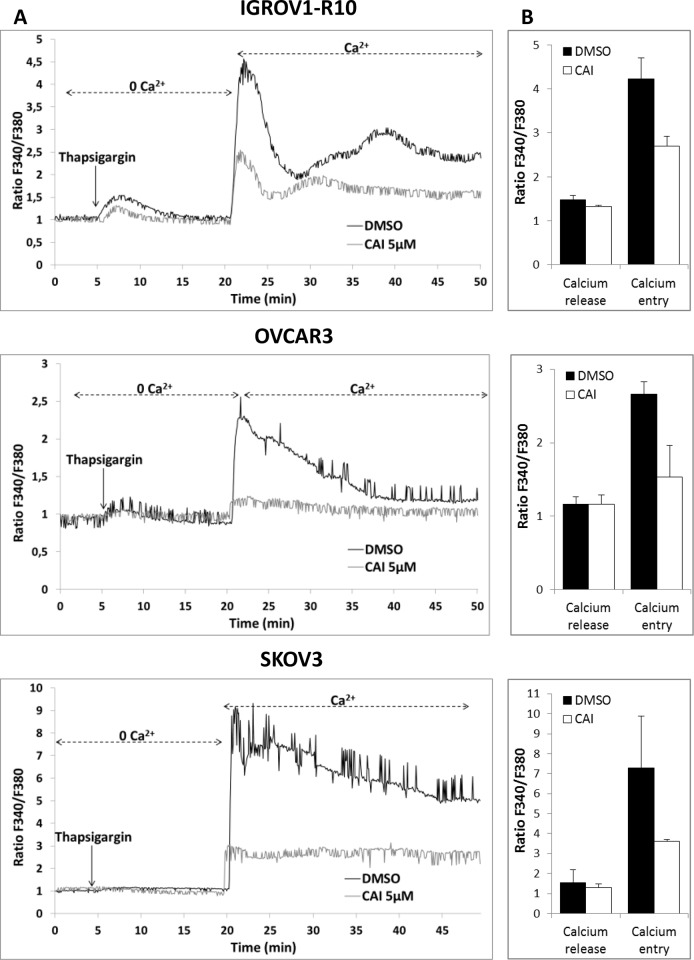
CAI does not block IP3R calcium release but inhibits SOCE **(A)** Effect of CAI on IP3R and SOC. Microspectrofluorimetry using Fura-2AM probe was performed in the three carcinoma cell lines IGROV1-R10, OVCAR3 and SKOV3 cells. During exposure to 0Ca^2+^, depletion of the intracellular stores was triggered by the addition of 2 μM thapsigargin to the bathing medium. Subsequent replenishment of 2 mM Ca^2+^ to the medium elicited a rise in [Ca^2+^]_i_ due to Ca^2+^ influx through open SOC. Black tracings depict the representative changes in [Ca^2+^]_i_ recorded from DMSO-treated cells and grey tracings depict the representative changes in [Ca^2+^]_i_ recorded from cells pre-treated for 1h with 5μM CAI. **(B)** Means ± SEM of the peaks of thapsigargin-induced Ca^2+^ release and Ca^2+^ store-operated channel entry recorded from CAI pre-treated cells (black bar) or not (DMSO) (white bar) (n=3).

Cells were thus placed in a Ca^2+^-free HBBSS medium with 2 μM thapsigargin, an inhibitor of sarcoplasmic-endoplasmic reticulum Ca^2+^-ATPase (SERCA), in order to deplete calcium from the endoplasmic reticulum. Upon this treatment, IGROV1-R10, OVCAR3 and SKOV3 cells exhibited a rapid rise in [Ca^2+^]_i_ reflecting calcium release from RE *via* the IP3 receptor [52]. Subsequent addition of 2 mM CaCl_2_ to the extracellular medium triggered a sustained increase in [Ca^2+^]_i_ from baseline (x4.23 in IGROV1-R10, x2.66 in OVCAR3 and x7.28 in SKOV3), which is consistent with a characteristic SOCE-mediated Ca^2+^ influx from the extracellular solution (Figure 2A and 2B). The experiment reiterated with cells pretreated for 1 hour with 5 μM CAI showed that whereas CAI did not inhibit calcium release from IP3R, it potently inhibited SOCE in the three cell lines (Figure 2A and 2B). Altogether, this strongly suggests that CAI inhibits store-operated calcium channels in ovarian carcinoma cells.

### CAI-induced Mcl-1 inhibition is not transcriptional or post-translational but is correlated with inhibition of mTORC1 pathway

**Figure 3:**
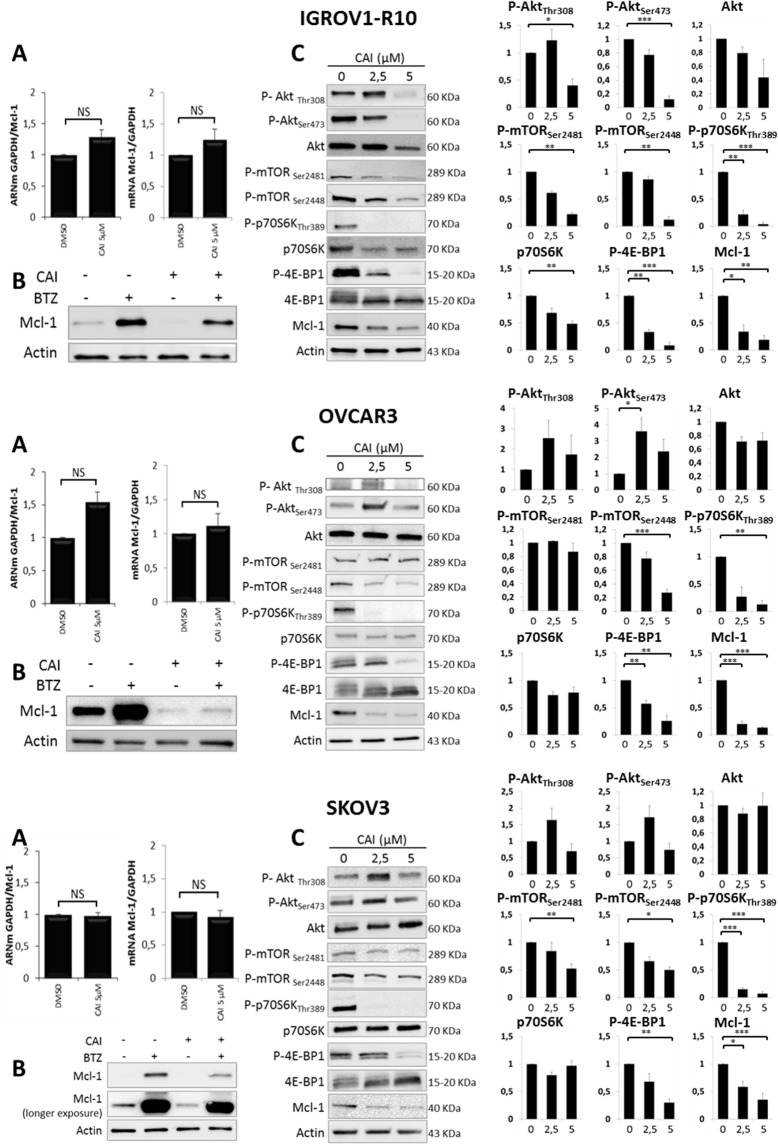
Mechanisms involved in CAI-induced Mcl-1 decrease **(A)** Cells were treated or not (DMSO) with 5 μM CAI for 24h (left panel) and 48h (right panel) for IGROV1-R10 and 48h (left panel) and 72h (right panel) for OVCAR3 and SKOV3. Mcl-1 mRNA expression was assessed by real time quantitative RT-PCR. GAPDH was used as a housekeeping reference gene for normalization. Histograms represent the relative mRNA expression in treated cells normalized to that of control cells. Data are expressed as mean ± SEM of three independent experiments. Statistical differences were analyzed with a Student t-test: NS=non-significant; **(B)** Ovarian carcinoma cell lines were treated or not (DMSO) with 5 μM CAI for 48h for IGROV1-R10 and 72h for OVCAR3 and SKOV3. One hundred nM bortezomib were added for the last 24h of treatment. Mcl-1 expression was monitored by western blot; **(C)** Cells were treated with increasing dose of CAI for 48h for IGROV1-R10 and 72h for OVCAR3 and SKOV3. The effect of CAI treatment on the activation of the PI3K/Akt/mTOR pathway was analyzed by studying the protein expression of P-Akt (Ser473 and Thr308) and total Akt, P-mTORC1 (Ser2448) and P-mTORC2 (Ser2448), P-p70S6K (Thr389) and total p70S6K and P-4E-BP1 (Thr70) and total 4E-BP1 by western blot analysis. Protein levels (standardized based on actin) were determined by densitometry scanning with Image J software to generate the values shown in the bar graphs. Results are expressed as mean ± SEM. Statistical differences were analyzed with a Student t-test: ^*^p< 0.05, ^**^p< 0.01, ^***^p< 0.001 (n=3).

To decipher the mechanisms underlying Mcl-1 down-regulation in response to CAI, Mcl-1 mRNA expression was quantified by using RT-qPCR. Treatment of cells with 5 μM CAI for 24h and 48h (IGROV1-R10) or 48h and 72h (OVCAR3 and SKOV3) did not inhibit Mcl-1 expression at mRNA level (Figure 3A), suggesting that CAI induced Mcl-1 down-regulation through transcription-independent mechanisms.

To investigate whether the Mcl-1 decrease upon CAI treatment was a consequence of proteasomal degradation, we incubated ovarian carcinoma cells with CAI for 48h (IGROV1-R10) or 72h (OVCAR3 and SKOV3) but supplemented the medium with bortezomib, a proteasome inhibitor, for the last 24 hours before analysis. As depicted in Figure 3B, bortezomib alone inhibited Mcl-1 degradation, which led to a strong up-regulation of Mcl-1 protein expression in the three cell lines tested. However, when CAI was added, bortezomib did not prevent the loss of Mcl-1 protein triggered by CAI. This suggests that CAI inhibits Mcl-1 protein through mechanisms upstream of proteasomal degradation, perhaps via translational events.

Therefore, we assessed the impact of CAI on the Akt/mTOR pathway, which is known to control Mcl-1 translation. Treatment with CAI dose-dependently inhibited mTORC1 activation in the three cell lines, as reflected by a decrease in P-mTORC1(Ser2448), P-4E-BP1 and P-p70S6K (Figure 3C). In agreement with previous reports, P-mTORC1(Ser2448) inhibition led to a feedback loop that triggered a transient up-regulation of P-Akt(Thr308) and P-Akt(Ser473) [53, 54], an effect that was observed in OVCAR3 and SKOV3 cell lines. Regarding IGROV1-R10, although P-Akt(Thr308) was also up-regulated, CAI treatment dose-dependently inhibited P-Akt(Ser473), suggesting that it inhibits both mTORC2 and P-Akt(Ser473) [53]. Thus, despite context-dependent effects on Akt activation, these results suggest that CAI down-regulates Mcl-1 translation through mTORC1 inhibition in ovarian carcinoma cells.

We then investigated the molecular link between SOCE inhibition and the decrease in mTORC1 phosphorylation. For this purpose, we evaluated the involvement of CamKII, a calcium/calmodulin-stimulated serine/threonine protein kinase acting as one of the major calcium sensors in the cell. CamKII was described to modulate several cellular processes such as carcinogenesis by controlling cancer cell survival including mTOR activation [55, 56]. Phosphorylated CamKII (Thr286) was expressed in the three cell lines at basal level, but CAI did not inhibit its activation (Supplementary Data 1), suggesting that CamKII is not involved in Mcl-1 inhibition induced by SOCE down-regulation.

Moreover, mTORC1 is known to be negatively controlled by phosphorylated AMP-activated protein kinase (p-AMPK) [57]. As CAI inhibited mTORC1, we then tested whether it was able to induce AMPK activation in our models. However, as illustrated in Supplementary Data 1, CAI did not trigger AMPK activation in the three cell lines, suggesting that AMPK was not involved in mTORC1 down-regulation and that further investigation was required to elucidate the molecular pathway involved.

### YM48583, a SOCE inhibitor, down-regulates mTORC1 targets and Mcl-1

**Figure 4:**
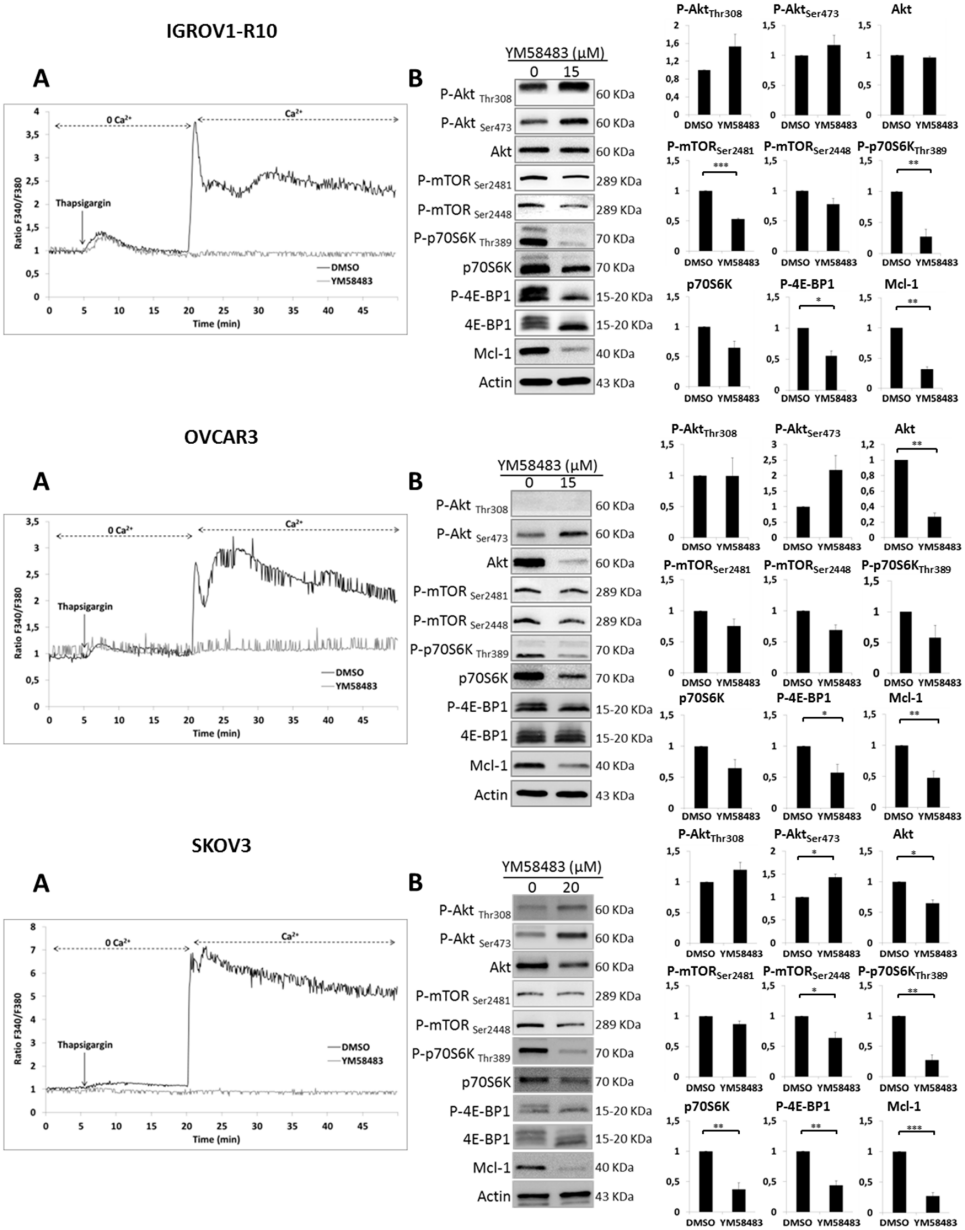
YM48583 blocks SOCE and inhibits mTORC1 targets and Mcl-1 **(A)** Effect of YM58483 on IP3R and SOC. Microspectrofluorimetry using Fura-2AM probe was performed in the three carcinoma cell lines IGROV1-R10, OVCAR3 and SKOV3 cells. During exposure to 0Ca^2+^, depletion of the intracellular stores was triggered by the addition of 2 μM thapsigargin to the bathing medium. Subsequent replenishment of 2 mM Ca^2+^ to the medium elicited a rise in [Ca^2+^]_i_ due to Ca^2+^ influx through open store-operated channels. Black tracings depict the representative changes in [Ca^2+^]_i_ recorded from DMSO treated cells and grey tracings depict the representative changes in [Ca^2+^]_i_ recorded from cells pre-treated 1h with 15 μM for IGROV1-R10 and OVCAR3 cells or 20 μM for SKOV3 cells (data are representative of three independent experiments). **(B)** Effect of YM58483 on Akt/mTORC1 pathway. Cells were treated with YM58483 (15 μM for 48h for IGROV1-R10 cells, 15 μM for 72h for OVCAR3 cells and 20 μM for 72h for SKOV3 cells). The effect of YM58483 treatment on the activation of the PI3K/Akt/mTOR pathway was analyzed by studying the protein expression of P-Akt (Ser473 and Thr308) and total Akt, P-mTORC1 (Ser2448) and P-mTORC2 (Ser2448), P-p70S6K (Thr389) and total p70S6K and P-4E-BP1 (Thr70) and total 4E-BP1 by western blot analysis. Protein levels (standardized based on actin) were determined by densitometry scanning with Image J software to generate the values shown in the bar graphs. Results are expressed as mean ± SEM. Statistical differences were analyzed with a Student t-test: ^*^p< 0.05, ^**^p< 0.01, ^***^p< 0.001 (n=3).

The involvement of SOCE in Mcl-1 down-regulation was confirmed using YM58483, a potent SOCE inhibitor [58]. We first verified that YM58483 was able to inhibit SOCE in our models. For this purpose, a “Ca^2+^ re-addition” protocol was used in the three cell lines pre-treated or not with YM53483. YM53483 did not inhibit calcium release triggered by thapsigargin in Ca^2+^ depleted medium but, as expected, it strongly reduced calcium entry *via* SOCE upon calcium reintroduction (Figure 4A). We then investigated whether YM58483 would mimic the effect of CAI on mTORC1 and Mcl-1 expression. For this purpose, we treated the cells for 48h (IGROV1-R10) and 72h (OVCAR3 and SKOV3) with YM53483 and assessed protein expression by western blot. As depicted in Figure 4B, YM53483 decreased phosphorylation of mTORC1(Ser2448) and its downstream targets, P-4E-BP1, P-p70S6K as well as Mcl-1 expression in the three cell lines tested. It did not down-regulate phosphorylation of Akt at residues Thr308 and Ser473, but through mTORC1 inhibition, released loops that re-induce their activation, as suggested by the literature [53]. Finally, YM58843 neither deactivated CamKII nor induced AMPK phosphorylation, strongly suggesting that inhibiting SOCE down-regulates Mcl-1 through mTORC1 inhibition (Supplemantary Data 2).

### SOCE inhibition by CAI or YM58483 combined with anti-Bcl-x_L_ strategies leads to apoptosis in ovarian carcinoma

**Figure 5:**
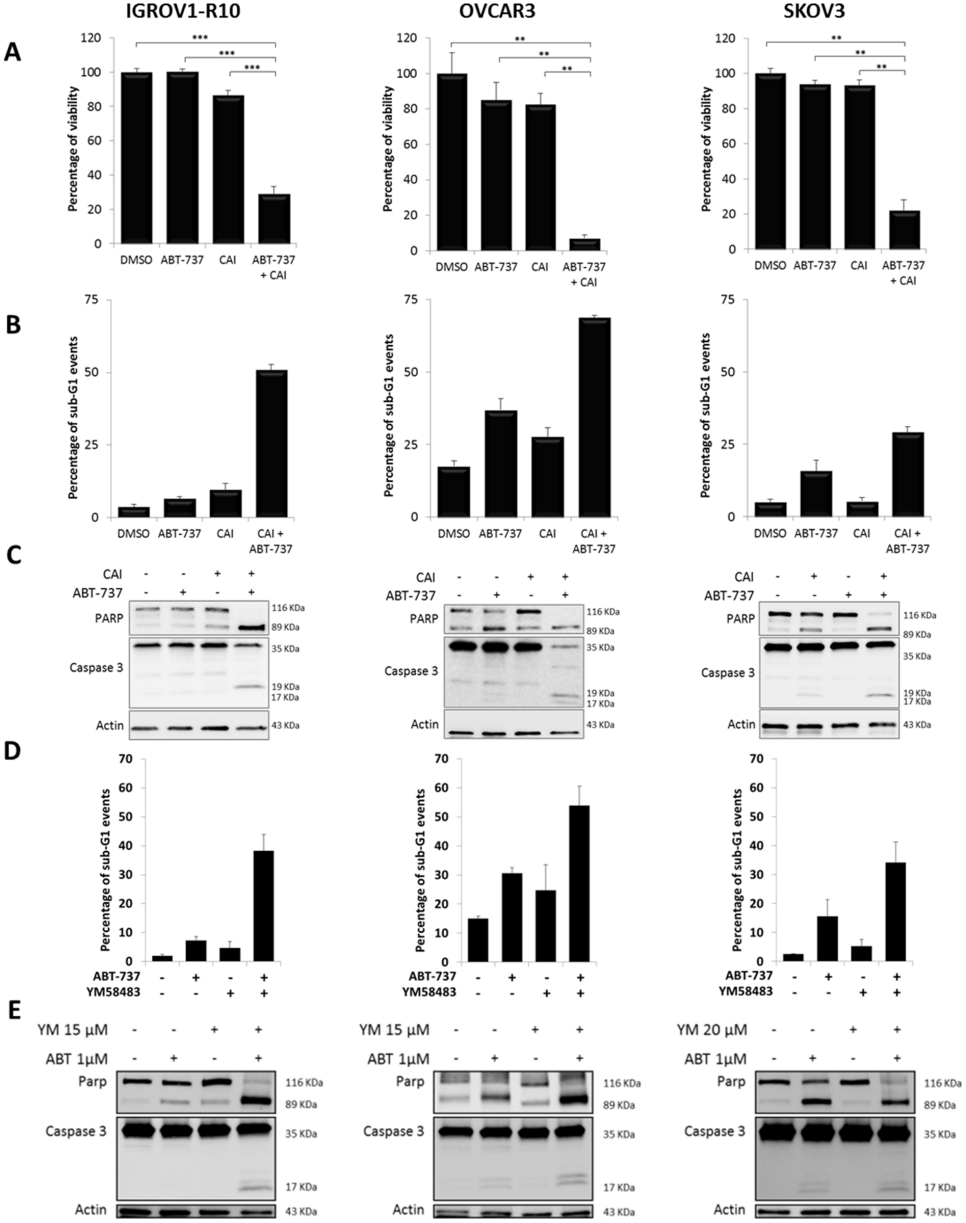
CAI or YM58483 combined with ABT-737 leads to apoptosis in ovarian carcinoma Cells were treated or not (DMSO) with 5 μM CAI for 48h for IGROV1-R10 or 72h for OVCAR3 and SKOV3, and 1 μM ABT-737 was added 24h before the end of the experiment. **(A)** Cell viability was assessed by trypan blue exclusion. Data are expressed as mean ± SEM of three independent experiments. Statistical differences were analyzed with a Wilcoxon test: ^*^p< 0.05, ^**^p< 0.01, ^***^p< 0.001. **(B)** Sub-G1 events were studied by flow cytometry. Data are expressed as mean ± SEM of three independent experiments. **(C)** PARP and caspase 3 cleavages were assessed by western blot. **(D)** YM58483 sensitizes ovarian carcinoma cells to ABT-737. Cells were treated with YM58483 (15 μM for 48h for IGROV1-R10 cells, 15 μM for 72h for OVCAR3 cells and 20 μM for 72h for SKOV3 cells). One micromolar ABT-737 was added for the last 24h hours of treatment. Sub-G1 events were studied by flow cytometry. **(E)** YM58483 sensitizes ovarian carcinoma cells to ABT-737. Cells were treated with YM58483 (15 μM for 48h for IGROV1-R10 cells, 15 μM for 72h for OVCAR3 cells and 20 μM for 72h for SKOV3 cells). One micromolar ABT-737 was added for the last 24h hours of treatment. PARP and caspase 3 cleavages were assessed by western blot.

As Bcl-x_L_ and Mcl-1 cooperate to protect ovarian carcinoma cells from apoptosis, we next evaluated the efficacy of SOCE inhibitor/anti-Bclx_L_ strategies. First, we combined CAI with the BH3-mimetic ABT-737. As CAI-induced modulation of protein expression occurred more slowly than ABT-737-inhibited Bcl-x_L_ activity, cells were pretreated with CAI for 24h (IGROV1-R10 cells) or 48h (OVCAR3 and SKOV3 cells). Then the medium was supplemented with 1μM ABT-737 for the last 24h of treatment. As illustrated in Figure 5A, this combination led to a drastic decrease in cell viability of the three cell lines (about −75% for IGROV1-R10, −90% for OVCAR3 and −80% for SKOV3 cells). This observation was supported by the potent increase in sub-G1 peaks on flow cytometry (Figure 5B), as well as by the occurrence of PARP and caspase 3 cleavages on western blotting (Figure 5C). Moreover, combining CAI with siRNA targeting Bcl-x_L_ triggered massive apoptosis in the three cell lines, as suggested by caspase 3 and PARP cleavages (Supplementary Data 3).

We then evaluated the efficacy of the YM58483/ABT-737 combination in our carcinoma cell lines. Whereas YM58483 did not induce any cell death on its own at the concentrations tested, its combination with ABT-737 elicited a massive apoptosis, as displayed by the emergence of a strong sub-G1 peak and caspase 3 and PARP cleavages (Figure 5D and 5E). Altogether, these experiments illustrate that inhibiting SOCE strongly sensitizes chemoresistant ovarian cells to anti-Bcl-x_L_ strategies.

## DISCUSSION

Despite remarkable progress in the understanding of apoptosis resistance, no alternative to platinum-based therapy has been approved yet for ovarian cancer. Hence, the development of new therapeutic strategies represents a crucial challenge for improving the present low survival rate of patients with advanced ovarian cancer. Several studies have shown that the anti-apoptotic proteins Bcl-x_L_ and Mcl-1 could constitute key targets for future therapeutic strategies [7, 59, 60]. In this line, we previously demonstrated that calcium signaling modulation targeted Mcl-1 translation and could be used to sensitize ovarian carcinoma to anti-Bcl-x_L_ strategies [13]. Our objective was then to test the effect of CAI, a calcium inhibitor used in clinical trials, on Mcl-1 expression and to evaluate its ability to sensitize ovarian carcinoma cells to anti-Bcl-x_L_ strategies.

CAI elicited an anti-proliferative effect in the three ovarian cancer cell lines tested, an effect consistent with several studies in other types of cancers at the same range of CAI concentrations and for the same durations of treatment [36, 37, 39, 61–63]. In our conditions, this effect was accompanied neither by a G2/M cell cycle arrest nor any apoptotic features, as observed in other cancer cell lines [63–66]. This could be due to the higher CAI concentrations (30 μM instead of 5 μM) or longer treatment times used in these studies. This could also explain why in our conditions Bcl-2 was not decreased upon CAI, as was the case in the MCF-7 breast cancer cell line [64]. In our hands, CAI potently inhibited Mcl-1 expression in ovarian carcinoma cells. Its effect cannot be ascribed to caspase 3 cleavage so its inhibitory effect is not a consequence of cell death, as already described [49]. This result is supported by our previous finding that Mcl-1 is decreased upon calcium signaling inhibition. To our knowledge, this is the first time that Mcl-1 has been found to be regulated by CAI in cancer cell lines.

CAI drastically inhibited SOC in our experimental models suggesting that it acts as a potent SOC inhibitor in ovarian carcinoma cells. SOC is one of the major calcium channels in non-excitable cells. These channels are composed of two sub-units, one belonging to the ER membrane (STIM-1) and the other to the cell plasma membrane (ORAI-1). Upon ER calcium depletion, these subunits interact with each other leading to a calcium influx from extracellular compartments in order to refill cytoplasm and ER stocks subsequently. Our finding is supported by other studies showing that CAI inhibits SOC in HEK293 [40], colon carcinoma cells [33], human endothelial cells [34] and endothelial progenitor cells isolated from patients [39]. In contrast, it did not significantly inhibit calcium release from IP3 receptor induced by thapsigargin, as observed in HUVEC and Rat-1 cells [67].

CAI-induced Mcl-1 inhibition did not require transcriptional events and its effect was not reversed by proteasomal inhibition, implying that post-translational events were not involved in the Mcl-1 decrease. The Mcl-1 down-regulation we evidenced may be attributed to translational events, as CAI strongly repressed P-4E-BP1, which is known to control Mcl-1 protein expression [11, 12]. This result corroborates those obtained with the calcium chelator BAPTA-AM in the same experimental models. Indeed, we previously found that BAPTA-AM treatment led to down-regulation of Mcl-1, which was counteracted by the overexpression of the 4E-BP1 target (eiF4E) [13].

Calcium can control the PI3K-AKT-mTOR pathway at several levels”. Indeed, calcium/calmodulin(CaM) complex has been shown to activate P-Akt(Ser473), and subsequently mTORC1, through CaMKII activation in B lymphocytes [30]. Consistent with this finding, SKF-96365, another SOCE inhibitor, inhibited the calcium/CaMKIIγ/Akt/mTORC1-mediated pathway in colorectal cancer cells [55]. However, CAI did not inhibit the activation of CaMKII in our carcinoma cell model. Other studies reported that calcium could activate this survival pathway through a calcium/CaM/CaMKK2 complex that bound to and activated P-Akt(Thr308) in ovarian carcinoma cells [68]. Nevertheless, in our conditions, P-Akt(308) was not down-regulated by CAI. Furthermore, since one of the major targets of CamKK2 is AMPK protein, it could be hypothesized that decreasing calcium could lead to a down-regulation in the CamKK2/AMPK pathway and consequently to an up-regulation of mTORC1, as previously described [69, 70]. Nonetheless, the contrary result we obtained implies that calcium/CamKK2 is not the main pathway targeted by CAI and that CamKK2 is not involved in mTORC1 and Mcl-1 down-regulation.

Our results strongly suggest that SOCE inhibition by CAI impaired PI3K/Akt/mTOR by inactivating mTORC1, as evidenced by the potent inhibition of mTORC1(Ser2448), p70S6K and 4E-BP1 protein phosphorylation. This effect was confirmed by the appearance of a transient upregulation of P-Akt(308) triggered by a compensatory feedback upon mTORC1 inhibition [54]. It is noteworthy that in OVCAR3 and SKOV3 cells, this feedback loop also led to a transient up-regulation of P-Akt(473) due to the release of the inhibitory effect of mTORC1 on mTORC2, which phosphorylates Akt(473). However, this effect was not observed in the IGROV1-R10 cell line, suggesting that mTORC1 inhibition could lead to independent regulation of the two phosphorylation sites. This conclusion is supported by several studies showing that Akt(308) and Akt(473) reactivation depends on the cellular context [53].

The hypothesis that calcium could play an important role in the regulation of mTOR signaling was evidenced by the need for calcium to activate p70S6K [24–26]. Thereafter, the role of calcium and calmodulin in mTORC1 activation was observed in a context of amino acid starvation [27]. Finally, lysosomal calcium was demonstrated to activate mTORC1 by inducing an association of calmodulin with mTORC1; the authors suggested that mTORC1 could be regarded as an “atypical calmodulin-dependent kinase” [31]. They also showed that although calcium signaling repression inhibited both mTORC1 and mTORC2, mTORC1 was inhibited at lower concentrations than those at which mTORC2 was inhibited. This supports our finding that calcium inhibition could down-regulate mTORC1 activation independently of its action on Akt(473).

To consolidate our findings with CAI, we then tested the effect of YM-58483, a well-known SOC inhibitor, on SOC channels and Akt/mTOR activation in ovarian carcinoma cells. As expected, YM58483 strongly down-regulated SOCE but had no impact on calcium release from IP3R, thereby confirming its action as an SOCE inhibitor. Moreover, its effect on the PI3K/Akt/mTOR pathway mimicked that obtained with CAI. YM85483 potently inhibited mTORC1 activation, leading to compensatory feedback loops on Akt and to inhibition of the mTORC1 downstream targets P-p70S6K and P-4E-BP1. Finally, YM-58483 also drastically inhibited Mcl-1 expression, so it is highly likely that Mcl-1 is a target for SOCE inhibitors. The link between SOCE and the expression of Bcl-2 family members had already been pointed out by a few studies but led to divergent conclusions. On the one hand, a constitutively active mutated STIM-1 led to induction of Mcl-1 transcription in HEK293; on the other hand, ORAI1 knock-down promoted Mcl-1 and inhibited Noxa expressions leading to survival of activated murine T cells [71, 72]. These discrepancies are probably inherent to the experimental model so further investigations are required to unravel the molecular pathway involved.

SOC play a crucial role in tumor development and accumulating evidence suggests that their activation is involved in cancer progression. First, they are differentially expressed in malignant cells and STIM1 and ORAI1 were found to be more strongly expressed in hepatoma tissues than in pre-cancerous tissues or non-tumoral tissues from the same patients [73, 74]. Second, they were demonstrated to play a crucial role in the invasion of glioblastoma cells [75], in angiogenesis by endothelial progenitor cells isolated from tumoral patients [39] and in migration and metastasis of breast cancer [76] and cervical cancer cells [77]. Finally, SOCE inhibition sensitized various cancer cells to apoptosis. It potentiated 5-FU-induced inhibition of the PI3K/AKT/mTOR pathway and synergized with 5-FU to induce cell death [74]. Furthermore, SKF96365 potentiated the anticancer effect of hydroxychloroquine in a mouse xenograft model through calcium/CaMKIIγ/AKT/mTOR inhibition [55]. Finally, in ovarian carcinoma, STIM and ORAI1 were found to be more strongly expressed in cisplatin-resistant cells (A2780cis) than in their sensitive counterpart (A2780), and the combination of cisplatin with 2-APB, a SOC inhibitor, restored A2780cis sensitivity [78]. Taken together, these findings suggest that SOC could provide interesting predictive factors of cancer progression and that targeting them could sensitize carcinoma cells to apoptosis.

On the basis of these results and those we previously obtained with BAPTA-AM [13], we combined CAI or YM58483 with the BH3-mimetic ABT-737 and found that they strongly sensitized ovarian carcinoma cell lines to anti-Bcl-x_L_ strategies in the three cell lines tested. Because our experimental model is dependent on Mcl-1 and Bcl-x_L_ to survive, the massive cell death we observed with SOC inhibitors and an inhibitor of Bcl-x_L_ strongly suggests that Mcl-1 expression is regulated by SOC channels. Moreover, the anti-tumoral effect of this combination could explain the mild efficacy of CAI when it was used as a single agent in clinical trials, as the sole inhibition of Mcl-1 is not sufficient to induce apoptosis in ovarian cancer [45]. CAI has been tested in combination with several compounds and has been shown to potentiate the anti-tumoral activity of sorafenib [63], LM-1685, a celecoxib analogue [79], 2-deoxyglucose [80] and Temozolomide [48]. In this recent phase IB clinical trial conducted in cohorts of recurrent or newly diagnosed glioblastoma, patients were treated with increasing doses of the oral derivative of this calcium channel blocker (Carboxyamidotriazole orotate - CTO) in combination with temozolomide ± radiotherapy. The results showed, on the one hand, that CTO is well tolerated by patients confirming results observed in previous clinical trials and on the other hand that treatment was effective on these aggressive cancers. This clinical trial showed that CAI has to be combined with another anti-neoplastic molecule in order to have a good therapeutic efficacy and it should be noted that the best clinical responses were obtained for tumors with EGFR amplification as well as mutations of the Akt / mTOR, PTEN and PI3KCA pathway [48]. Therefore these results strongly support all those we have obtained in ovarian cancer.

To our knowledge, this is the first time that CAI has been shown to sensitize cancerous cells to Bcl-x_L_ inhibitors. Furthermore, this is the first report of SOC inhibitors sensitizing chemoresistant ovarian cells to anti-Bcl-x_L_ strategies. This offers interesting perspectives for the clinical use of ABT-263 (Navitoclax, the orally available ABT-737 analogue). Due to its ability to antagonize the survival function of Bcl-x_L_ in platelets, it triggers thrombocytopenia, which is its major dose-limiting side-effect. As CAI synergizes with ABT-737, it might allow a dose reduction of ABT-263, thereby alleviating the BH3-mimetic side-effect.

In conclusion, we show that CAI has an anti-proliferative effect on ovarian cell lines and that it inhibits Mcl-1 expression through SOCE/mTORC1 inhibition, which leads to a strong sensitization of ovarian carcinoma to anti-Bcl-x_L_ strategies. We hypothesize that CAI, *via* its action on SOCE and Mcl-1, could extend the therapeutic arsenal for ovarian cancer treatment if combined pertinently. As SOC are known to interact with the frequently deregulated PI3K/Akt/mTOR pathway, they could offer a relevant target to overcome apoptosis resistance in ovarian carcinoma.

## MATERIALS AND METHODS

### Cell culture

The human platinum-resistant ovarian carcinoma cell lines IGROV1-R10, OVCAR3, and SKOV3 were used. IGROV1-R10 was established as described previously (34) from the IGROV1 cell line, kindly provided by Dr. Jean Bénard (Institut Gustave Roussy, Villejuif, France). OVCAR3 and SKOV3 were obtained from the ATCC. The cell lines were authenticated in April 2016 by Microsynth who compared their STR profiles with the ATCC database. They were grown in RPMI1640 medium (Gibco) supplemented with 2 mM Glutamax™, 25 mM HEPES, 10% FBS (Gibco) and 33 mM sodium bicarbonate and were maintained in a 5% CO_2_ humidified atmosphere at 37°C.

### Reagents

CAI, YM58493, thapsigargin were provided by Tocris (R&D Systems). ABT-737 was supplied by Selleckem and Bortezomib (Velcade®) was supplied by Millennium Pharmaceuticals. Fura-2AM was purchased from Thermo Fisher Scientific. These compounds were commonly stored as stock solutions in DMSO at −20°C.

### Proliferation analysis

Cell number and viability were estimated by a semi-automated image-based cell analyzer (Cedex XS Analyzer, Roche Applied Science, Meylan, France) using the Trypan blue exclusion method.

### Cell cycle analysis by flow cytometry

Adherent and floating cells were pooled, washed with phosphate-buffered saline (PBS 1X) and fixed with ethanol 70%. Cells were then centrifuged at 2000 rpm for 5 min and incubated for 30 min at 37°C in PBS 1X, to allow the release of low-molecular weight DNA. Cell pellets were stained with propidium iodide using the DNA Prep Coulter Reagent Kit (Beckman-Coulter). Samples were analyzed with a Gallios flow cytometer (Beckman Coulter).

### Western immunoblotting

Cells were rinsed with ice-cold PBS 1X and lysed in lysis buffer (15 mM HEPES, 50 mM KCl, 10 mM NaCl, 1 mM MgCl_2_, 0.25% glycerol, 0.5% laurylmaltoside, 5 μM GDP, 1 μM microcystin (Enzo Life Sciences), 1 mM sodium orthovanadate and cOmplete Protease Inhibitor Cocktail (Sigma-Aldrich/Roche). After centrifugation, proteins were quantified using the Bradford assay (Bio-Rad, CA). Thirty micrograms of proteins were separated by SDS–PAGE (Biorad) and transferred to PVDF membranes (Millipore). After blocking, membranes were incubated overnight at 4°C with the following primary antibodies: Bcl-2 (#M0887, DAKO), actin (MAB1501, Merck Millipore), Mcl-1 (#5453), Bcl-x_L_ (#2764), PARP(#9542), caspase-3 (#9662), P-Akt (Thr308; #13038), P-Akt (Ser473; #4060), Akt (#9272), P-4E-BP1 (Thr70; #4370), 4E-BP1 (#9644), P-p70S6K (Thr389; #9205) and p70S6K (#9202), P-mTOR (Ser2448; #5536), P-mTOR (Ser2481; #2974), p-CamKII (Thr286; #12716), p-AMPK (Ser485; #4184), AMPK (#2532) (Cell Signaling Technology). Membranes were then incubated with the appropriate horseradish peroxidase-conjugated secondary antibodies (GE Healthcare). Revelation was done using Clarity Western ECL (Biorad). Western blots shown are from one experiment representative of at least three independent experiments and cell lysates. Signals were quantified by pixel densitometry using the ImageJ software.

### RNA extraction and real-time quantitative reverse transcription PCR (qRT-PCR)

Total RNA was isolated from ovarian carcinoma cell lines using Trizol (Invitrogen, Life Technologies). RNA quantity and quality were assessed using the NanoDropTM 2000 spectrophotometer (Thermo Scientific). The first strand cDNA was synthesized using the Omniscript reverse transcriptase kit (Qiagen) with random hexamers. cDNA (25 ng) were combined with 10 μmol/l of each forward and reverse primer, 50 μmol/l of the Taq-Man® probe and TaqMan® Fast Universal PCR Master Mix (Applied Biosystems) in a 20 μl final reaction volume. Corresponding custom inventoried (ID: Hs00172036_m1 for Mcl-1 and Hs99999905_m1 for GAPDH) TaqMan® Gene Expression Assays were used (Applied Biosystems). All PCR amplification reactions were carried out in triplicate and detection was done on an Applied ABI Prism 7500 Fast PCR system (Applied Biosystems). GAPDH was used as a housekeeping reference gene for normalization.

### siRNA transfection

PAGE-purified siRNAs were synthesized and annealed by the Eurogentec Company. Specific double-stranded 21 nt RNA oligonucleotides forming a 19 bp duplex core with 2 nt 3′ overhangs were used to silence Bcl-x_L_ (5′ auuggugagucggaucgca-3′, noted siXL). siGENOME Non-Targeting siRNA Pool#1 (noted siCT) was purchased from Dharmacon. siRNA duplexes were transfected using the INTERFERin™ transfection reagent according to the manufacturer's instructions (Polyplus-Transfection). Briefly, cells were seeded in 25 cm^2^ flasks to reach 30–50% of confluence at the time of transfection. The transfection reagent and the siRNAs were mixed and complex formation was allowed to proceed for 15 min at room temperature before application to cells. After the indicated time, cells were trypsinized and washed with ice-cold PBS 1X before analysis.

### Calcium imaging

Intracellular calcium concentration variations were analyzed by microspectrofluorimetry using the Ca^2+^-sensitive probe Fura-2-AM as described previously [81]. Briefly, ovarian carcinoma cells were incubated at 37°C in HBBSS buffer (116 mM NaCl; 5.4 mM KCl; 0.8 mM MgSO_4_; 12 mM HEPES; 0.34 mM NaH_2_PO_4_; 25 mM NaHCO_3_; 5.5 mM D-glucose; 10 μM Glycine; 1.8 mM CaCl_2_; pH 7.4) supplemented with 10 μM Fura-2-AM (#F1201 Thermofisher Scientific) for 45 min. Following probe loading, cells were treated or not by 5 μM CAI for 45 min. Then they were placed in a recording chamber mounted on the stage of an epifluorescence inverted microscope (Lieca DMI 6000 B) equipped with a 150 W xenon high stability lamp and a Leica 40 ×, 1.3 numerical aperture epifluorescence oil immersion objective (Wetzlar, Germany). Fura-2AM-loaded cells were treated with 2 μM thapsigargin in HBBSS without CaCl_2_ (0Ca^2+^) to trigger calcium efflux from the RE. To test SOCE, calcium was reintroduced in the medium by using classical HBBSS (+Ca^2+^). During the acquisition, cells were irradiated alternately with 340 and 380 nm light, and fluorescence from the trapped dye was measured at 510 nm. The ratio of fluorescence intensities recorded after excitation at 340 nm (F340) and at 380 nm (F380) was used to estimate intracellular calcium concentrations ([Ca^2+^]_i_) and was acquired every 5 seconds with a digital CMOS camera (Hamamatsu, ORCA-Flash2.8 C11440-10C). The monochromator and the photometers allow emission and detection of fluorescence from ~12 cells in the field of view. Data analysis was performed using MM fluor software (Universal Imaging Corporation).

### Statistical analysis

The values are presented as means ± S.E.M. for at least three independent experiments. Student's *t*-test was used for statistical analysis of densitometry graphs and Wilcoxon test was used for analysis of percentage of viable cells. Differences were considered statistically different if *p*<0.05 (^*^); *p*<0.01 (^**^); *p*<0.001 (^***^).

## SUPPLEMENTARY MATERIALS



## Abbreviations

4E-BP1: eukaryotic translation initiation factor 4E (eIF4E)-binding protein
AMPK: AMP-activated protein kinase
[Ca^2+^]_i_: intracytosolic calcium concentration
CAI: Carboxyamidotriazole
CaM: Calmodulin
CamKII: Ca 2+/calmodulin-dependent protein kinase II
CamKK2: Calcium/calmodulin-dependent protein kinase kinase 2
IP3R: Inositol Trisphosphate Receptor
mTORC1/2: mammalian Target Of Rapamycin Complex 1/2
p70S6K: p70 S6 kinase
PARP: Poly(ADP-ribosyl) polymerase
PI3K: phosphatidylinositol 3-kinase
STIM: stromal interaction molecule
ORAI: calcium release-activated calcium modulator
PDX: Patient derived xenograft
SERCA: sarco/endoplasmic reticulum Ca^2+^-ATPase
SOCE: Store-Operated Calcium Entry
TRP: Transient Receptor Potential.
