# Supplementary material for: Inhibition of store-operated channels by carboxyamidotriazole sensitizes ovarian carcinoma cells to anti-BclxL strategies through Mcl-1 down-regulation
Source: Oncotarget. 2018 Sep 21;9(74):33896–911. doi: 10.18632/oncotarget.26084 (PMC6188062; doi:10.18632/oncotarget.26084)
Supplement: Supplementary file 1 [file oncotarget-09-33896-s001.pdf]

Inhibition of store-operated channels by carboxyamidotriazole sensitizes ovarian carcinoma cells to anti-Bclx$_L$ strategies through Mcl-1 down-regulation

SUPPLEMENTARY MATERIALS

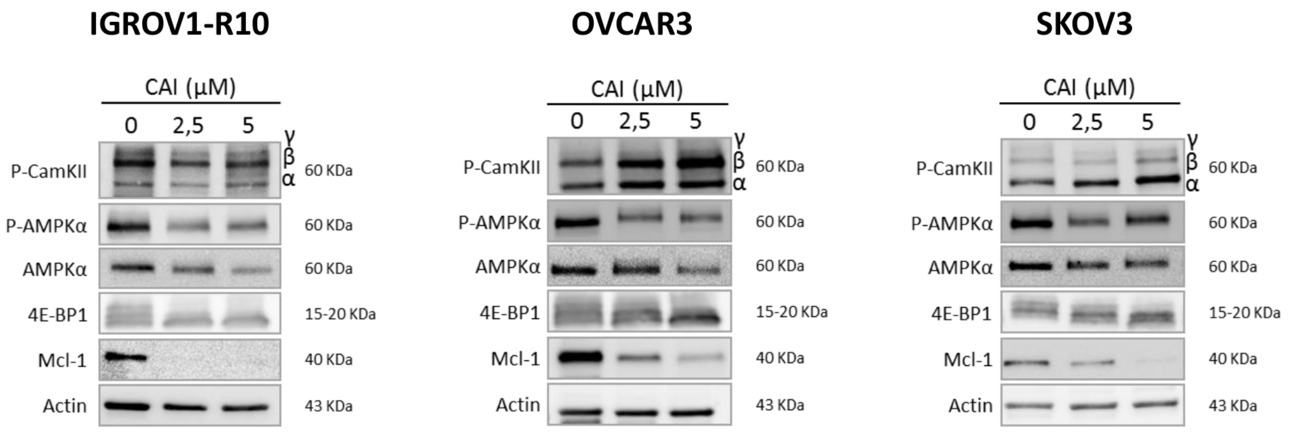

Supplementary Data 1: Inhibition of Mcl-1 by CAI does not involve CamKII and AMPK. Cells were treated with increasing doses of 5 μM CAI for 48h for IGROV1-R10 and 72h for OVCAR3 and SKOV3. The effect of CAI treatment on P-CamKII, P-AMPK, AMPK, 4E-BP1 and Mcl-1 was assessed by western blot.

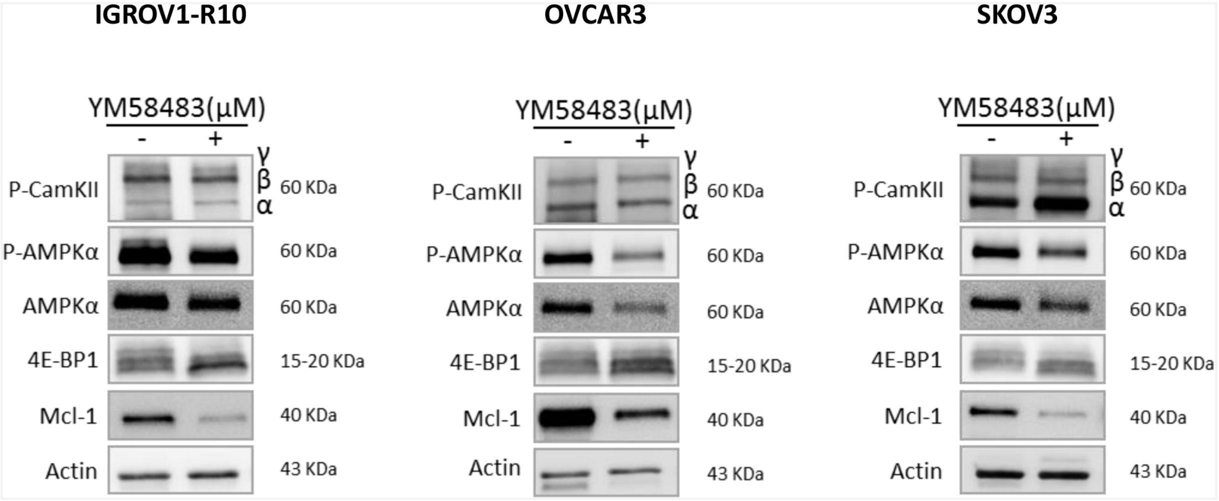

Supplementary Data 2: Inhibition of Mcl-1 by YM58483 does not involve CamKII and AMPK. Cells were treated with YM58483 (15 µM for 48h for IGROV1-R10 cells, 15 µM for 72h for OVCAR3 cells and 20 µM for 72h for SKOV3 cells). The effect of YM58483 treatment on P-CamKII, P-AMPK, AMPK, 4E-BP1 and Mcl-1 was assessed by western blot.

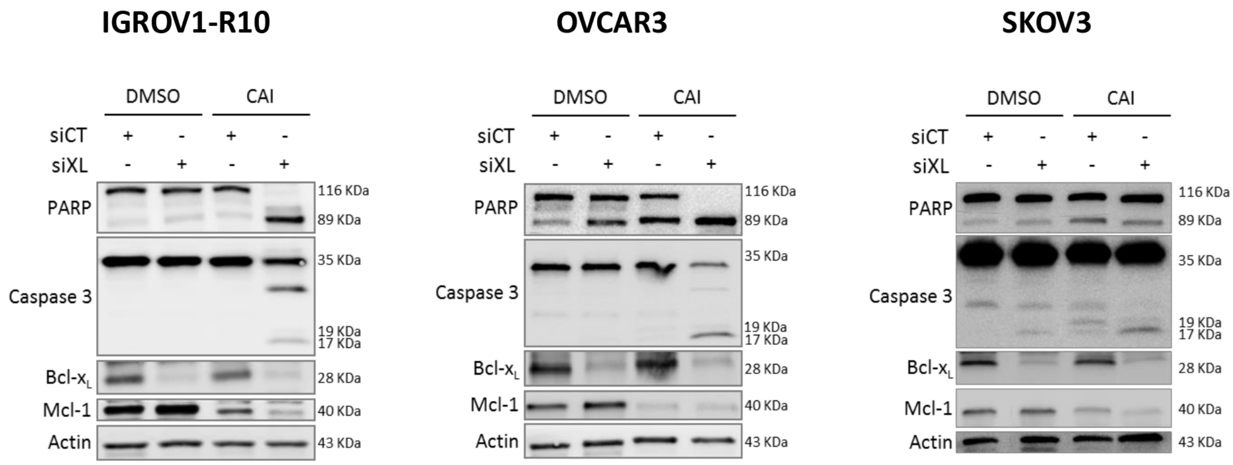

Supplementary Data 3: CAI combined with siRNA targeting Bcl-xL leads to apoptosis in ovarian carcinoma. Cells were transfected with anti-Bcl-x_L siRNA for 24h for IGROV1-R10 and OVCAR3 or 48h for SKOV3. Then cells were treated with 5 µM CAI for 48h for IGROV1-R10 and 72h for OVCAR3 or SKOV3. Mcl-1 and Bcl-x_L expressions and PARP and caspase 3 cleavages were studied by western blot.
